# The Impact of Relapses on Pain and Quality of Life in Patients with Multiple Sclerosis Treated with Corticosteroids

**DOI:** 10.3390/ph16091244

**Published:** 2023-09-04

**Authors:** Martin Rakusa, Jeremy Chataway, Todd A. Hardy

**Affiliations:** 1 Division of Neurology, University Medical Centre Maribor, Ljubljanska 5, 2000 Maribor, Slovenia; 2 Faculty of Medicine, University of Maribor, Taborska 8, 2000 Maribor, Slovenia; 3 Queen Square Multiple Sclerosis Centre, Department of Neuroinflammation, UCL Queen Square Institute of Neurology, Faculty of Brain Sciences, University College London, London WC1B 5EH, UK; 4 National Institute for Health Research, University College London Hospitals, Biomedical Research Centre, London W1T 7DN, UK; 5 Department of Neurology, Concord Hospital, University of Sydney, Sydney, NSW 2050, Australia; thar6109@sydney.edu.au; 6 Brain & Mind Centre, University of Sydney, Sydney, NSW 2050, Australia

**Keywords:** multiple sclerosis, relapse, pain, corticosteroids, quality of life, the short-form health survey, SF-36

## Abstract

Background: We assessed the prevalence and risks associated with pain during and after a multiple sclerosis (MS) relapse, and the impact of pain on quality of life (QoL), in MS patients. Methods: 117 patients suffering an acute MS relapse were evaluated with clinician- and patient-reported outcomes, including the expanded disability status scale (EDSS), Multiple Sclerosis Impact Scale (MSIS-29), and MS Walking scale-12 (MSWS-12). Relapse-related pain was assessed via the short-form 36 (SF-36) questionnaire upon first visit (relapse onset) and at 6 weeks after treatment with intravenous methylprednisolone (follow-up visit). Results: Pain was present in 80% of patients at relapse onset. Patients with pain were more impaired physically (higher mean scores on MSIS-29_phys_ and MSWS-12 and lower mean scores on SF-36 role physical, physical, and vitality scales) at relapse and six weeks after. In total, 74% of patients with MS relapse reported a poorer QoL due to pain. A lower psychological well-being was correlated with greater pain (MSIS29_psy_ score). An increased number of prior relapses was a predictor of more pain at relapse onset. Conclusions: Pain was common at the time of MS relapse and improved, but was still significant, six weeks after treatment with corticosteroids. Further studies are required to better understand relapse-related pain.

## 1. Introduction

Chronic pain is a common symptom in multiple sclerosis (MS). In one study that included patients with relapsing and progressive MS, 98% of patients reported bodily pain [1]. For some, it can be their dominant symptom, and it is a strong predictor of a reduced quality of life (QoL) [1,2,3,4,5].

Several studies have explored the risk factors for pain in patients with MS. In a systematic review of 21 studies, factors including female gender, non-relapsing-remitting MS type, a longer duration of MS, poor physical and emotional well-being, fatigue, and social factors (e.g., unemployment) were associated with chronic pain [6]. In the largest study, which included more than 1600 patients with MS, patients with a higher expanded disability status scale (EDSS) score had a greater likelihood of pain [7]. A similar association between pain and disability was found among patients in the German National MS Register [8], where there was a positive correlation between PainDETECT questionnaire score and EDSS, age, and duration of disease. However, in another smaller study of 120 patients, there was no correlation between pain, age, or duration of MS [4]. Although both studies evaluated neuropathic pain, the main difference between them were the sample sizes and questionnaires used. While in the German study they used PainDETECT [8], in the Lithuanian study, The Douleur Neuropathique 4 (DN-4) questionnaire was used [4]. The former is shorter and simpler in comparison to latter.

Few studies have looked at pain following MS relapses. Indeed, many studies on MS-related pain actively exclude patients during relapse. In a large systematic review and meta-analysis, Foley et al. analysed 28 papers studying pain in people with relapsing-remitting or secondary progressive MS [9]. Only one work included prospectively studied pain at relapse. Another study, which retrospectively evaluated the prevalence of pain in 73 patients, found that 63% of MS patients reported pain during relapse [10]. The Short-Form Health Survey (SF-36) is a self-reported quality of life (QoL) survey which is divided into psychometrically based physical and mental components [11]. It consists of 36 items covering 8 domains of patient health. Each domain evaluates four categories: function, well-being, disability, and personal evaluation. The Bodily Pain scale is considered part of the physical evaluation component and measures the severity and impact of pain. The SF-36 norms vary between ethnicity, gender, and age, but the mean score for body pain ranges between 50 and 80 out of 100 [12,13], with a low score on the SF-36 Bodily Pain scale indicating severe and limiting pain.

The aim of our study was to use the SF-36 patient-reported outcome measure to assess the prevalence and risks associated with pain both during an MS relapse and after treatment with corticosteroids, and the impact of pain on the QoL of MS patients.

## 2. Results

Data were collected for 117 patients (79% female) at the time of relapse with a mean age and SD of 38.3 (8.8) years. The mean SF-36 bodily pain scores between patients with and without pain significantly differed at relapse onset (37.6, CI [32.9–42.2] vs. 95.9, CI [93.9–98.5]; *p* < 0.001) and at 6-week follow-up (46.9, CI [19.9] vs. 93.7, CI [91.1–96.4], *p* < 0.001) (Table 1).

At relapse onset, 80% of the patients reported pain. More than half of the patients had moderate to very severe pain. Six weeks after relapse, the number of patients with moderate, severe, and very severe pain decreased, and the number of patients with no pain, very mild, and mild pain increased (Table 2).

For all patients, the EDSS, MSIS-29 (and sub-scales), and MSWS-12 scores were higher and the mean values on all the SF-36 scales were lower (more pain) at relapse onset than six weeks later (Table 1). At relapse onset, 74% of the patients reported an impaired quality of life due to pain and scored less than 80 points on SF-36. Six weeks after relapse onset, this pain had significantly decreased with an improvement in the quality of life of 65% of the patients. The mean change was significant (−9.5 points, SD 25.1, *p* value < 0.0001).

At relapse, patients with pain had higher mean scores on MSIS-29_phys_ and MSWS-12 and lower mean scores on the role physical, physical, pain, and vitality scales (Table 1). After relapse, pain was also associated with physical and mental status. Patients with pain scored less on all eight SF-36 scales and higher on the EDSS and MSIS-29 scales (Table 1).

### Correlation between Pain and Physical and Psychological Impairment

We found a significant association between more bodily pain and a greater physical and psychological impact at the time of the MS relapse and six weeks later (Table 3), as demonstrated by a moderate negative correlation between SF-36 Pain and the MSIS-29_PHY_, MSIS-29_PSY_, and MSWS-12 scores (*p* < 0.001).

Bodily pain was also significantly correlated with the other SF-36 variables. At relapse onset, a positive moderate correlation between pain and all the SF-36 variables was noted, except for emotional role functioning and general health perceptions, where the correlations were weak (*p* < 0.001). The correlations were stronger at the six-week follow-up than relapse onset. A moderately positive correlation was found between pain and all the SF-36 variables (*p* < 0.001).

## 3. Discussion

### 3.1. MS Relapses Were Associated with Pain

In this study, we explored the prevalence of pain in patients experiencing an MS relapse. The frequency of pain was high; that is, 80% reported pain at relapse onset (Table 2). Almost two thirds of these patients had moderate, severe, or very severe pain, and three quarters scored less than 80 points on the SF-36 Bodily Pain scale at relapse onset, indicating that their quality of life was impaired by pain. Six weeks after relapse, they reported less severe pain. The number of patients experiencing pain in the two groups was reduced by 6% to 8.5%, as well as an increased score being observed on the SF-36 Bodily Pain scale (Table 1 and Table 2).

The MS patients had worse pain at relapse and at 6 weeks relative to the general population in England [12]. The pain intensity after relapse was similar to that of a recent cross-sectional study on 431 patients with RRMS (independent of relapse), which reported 20% of patients being free of pain, 29% with mild pain, 31% with moderate pain, and 20% with severe pain [14]. Our patients had less pain 6 weeks after relapse onset than those in a recent German study that reported very mild background pain in 11.6%, mild pain in 16.2%, moderate pain in 37%, severe pain in 15.2%, and very severe pain in 4.1% of patients [1].

### 3.2. Worsening of Pain Is Associated with Worsening of Physical and Psychological Well-Being

Taken together, our results suggest that the worse the physical impairment at relapse, as evaluated by MSIS-29_PHY_ and MSWS-12 (but not EDSS), the worse the pain in people with MS. In most studies, EDSS does not correlate with the intensity of pain [15,16,17]. For example, Michalski et al. evaluated the severity and quality of pain in people with MS. and did not find significant differences in the EDSS scores of participants with and without pain. Tentuncu et al. studied people with relapsing-remitting MS and myofascial pain syndrome. They treated their pain with local anaesthetic injections. Three months after treatment, there were no differences in the EDSS scores between respondents and non-respondents [15]. In another study, Ate et al. studied neuropathic pain in people with relapsing-remitting MS and secondary progressive MS. Although half of the participants had neuropathic pain, there were no differences in the EDSS scores between people with and without neuropathic pain [17]. However, this finding is not universal, and some studies have demonstrated a moderate correlation between EDSS score and pain intensity [14]. However, their samples have consisted of people with relapsing-remitting and progressive MS who used a self-reported EDSS.

In addition to physical impairment, our results show an association between pain and a poorer psychological state measured either with MSIS-29_PSY_ or the mental domains of SF-36 (vitality, general health perceptions, social role functioning, mental health, and emotional role functioning). The scores in all of these domains significantly decreased in patients with more severe pain, both at relapse and six weeks later (Table 3).

In our study, demographic variables such as age and MS duration did not differ significantly between the patients with and without pain, at relapse onset or six weeks later, contrary to other studies [18], but in keeping with a recent study that did not find a correlation between age and pain intensity [14] and another study that did not demonstrate any differences in age or duration of MS between patients with and without pain [4].

We showed that patients who had more relapses also had more pain at relapse onset. At relapse onset, a weak positive correlation between the severity of pain and number of prior relapses was also found. Silva et al. demonstrated that relapse frequency may negatively correlate with chronic pain in women [19]. However, it is difficult to compare both studies directly, as, in their study, the incidence of pain was lower, and the patients had, on average, significantly more relapses than those in our group (2.8 vs. 2.03, respectively). Furthermore, these authors evaluated pain in general, independently of relapse.

### 3.3. Pain Significantly Impairs Quality of Life

Our results are in accordance with previous studies, which indicate that pain has an influence on all aspects of a patient’s life. Quality of life is significantly worse when patients score poorly on the SF-36 bodily pain, general health, role emotional, energy/vitality, mental health, and social functioning scales [20]. Patients with pain at relapse onset were more physically and psychologically impaired than patients without pain and had a lower quality of life in six areas measured with the SF-36 scales. Even after a relapse had physically remitted, the patients with pain continued to be more impaired than the patients without pain.

Similar to previous studies on chronic pain, we found a significant positive correlation between bodily pain and poorer scores across all SF-36 domains (Table 3), both at relapse onset and six weeks later. For patients with MS, pain could be a more important non-physical impairment for determining QoL than social functioning, vitality, or general and mental health [21]. The presence of pain is a fundamental factor that influences QoL [1]. Physical impairment influences several domains of QoL, such as bodily pain, general health, vitality, and social functioning [22]. Moreover, pain is strongly associated with the mental status of MS patients. MS patients with depression or fatigue have more pain than those without pain [1,23,24]. Janardhan and Bakshi studied people with relapsing-remitting or secondary progressive MS. The participants without depression achieved almost double scores in the Multiple Sclerosis Quality of Life-54 compared to those with depression. In another study, Tedman et al. assessed people with MS using the SF-36 and Hospital Anxiety and Depression Scale (HAD). They found a significant negative correlation between HAD depression subscore and the score on SF-36 Pain [24]. In a recent study, more than 400 people with relapsing-remitting or secondary progressive MS were examined using the Multiple Sclerosis Quality of Life-54 instrument [1]. Symptoms of depression and pain had a significant impact on patients’ quality of life.

The results from a recent survey in Australia demonstrated that depression and pain are the most important factors that negatively influence the QoL among MS patients [25]. That study did not differentiate between acute and chronic pain, or specify the types of pain (somatic/neuropathic), but it was notable that this relationship was not changed whether patients had relapsing or progressive forms of MS. A positive correlation between anxiety and pain may also exist in MS patients [5,24]. Labuz-Roszak et al. [26] evaluated 144 MS patients using HAD and the European Quality of Life-5 Dimensions (Euro-Qol 5D). Patients with current pain scored more on the HAD anxiety subscale than those without. Similar, Tedman et. al found a significant correlation between pain and HAD anxiety subscale score.

### 3.4. Limitations

Our study has several limitations. In the original trial from which these prospective data were taken, pain was not the major study endpoint and the 6-week duration of follow-up was limited. It was also not possible to differentiate between nociceptive and neuropathic pain, chronic versus acute relapse pain, or to identify pain syndromes that occur commonly in MS [6,27,28]. The patients were asked to estimate their pain over the preceding 4 weeks at each study visit, introducing the possibility of recall bias. As the study did not include an untreated control group, it was also not possible to draw conclusions about how much the improvement in pain following the MS relapse was dependent on corticosteroid treatment.

## 4. Materials and Methods

### 4.1. Patients

We analysed data collected as part of a previously published randomized controlled trial comparing the effectiveness of intravenous methylprednisolone (IVMP) at 1 g daily for 3 days given in an out-patient setting versus in an in-home setting, conducted at the National Hospital for Neurology and Neurosurgery, London, UK [29,30].

In the original trial, only patients with relapsing-remitting MS who did not have disease progression between relapses were included. They had acute MS relapses with a worsening of their neurological symptoms which lasted more than 24 h and less than 4 weeks. There were 149 eligible patients and 138 patients participated in the end. In the present study, we analysed the data from 117 adult MS patients; for 21 patients, we did not have information on their pain or the other analysed parameters. Therefore, we excluded them.

Patients were excluded if they had mild relapses defined by the treating physician as not having a significant effect on their function, or if they had relapses severe enough to require inpatient hospitalisation. Data on pain at the follow-up visit were not available for five patients. All the patients received corticosteroid treatment.

All the patients signed informed consent and the study was approved by the Ethics Committee of University College London Hospital, UK.

### 4.2. Clinical Evaluation

**Figure 1:**
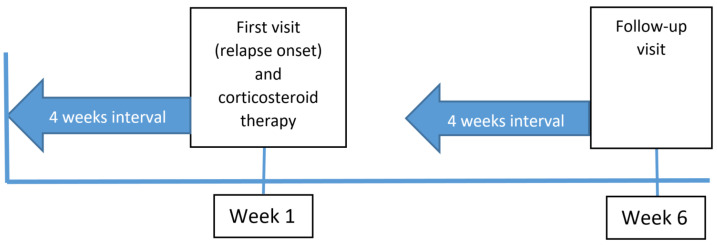
Evaluation of patients with SF-36 bodily pain scale. Patients attended their first visit when they had a relapse and estimated their level of pain retrospectively over the preceding four weeks using the SF-36 bodily pain scale. Patients were treated with three days of intravenous methylprednisolone 1 gram daily for their relapse and then attended for a follow-up visit six weeks later for re-assessment.

Data regarding pain were collected via the SF-36 bodily pain scale for the 4 weeks leading up to the first visit (relapse onset) and for the 4 weeks leading up to the post-corticosteroid visit at 6 weeks (follow-up visit) (Figure 1). 

Patients who scored less than 80 points on the SF-36 were considered to have an impaired quality of life due to pain, relative to the normative data established for the healthy population in England [12]. All the others were considered to be pain-free. The patients were divided into pain and pain-free groups according to their pain results at relapse onset and six weeks later (Table 1). The severity of their pain was defined according to the categories described in the SF-36 bodily pain domain questions.

The patients were also evaluated with the EDSS to assess their disability across eight functional systems: pyramidal, cerebellar, brainstem, sensory system, bowel/bladder function, visual function, and mental function. The patients were also assessed with the Multiple Sclerosis Impact Scale (MSIS-29), a patient-reported outcome scale that consists of two subscales, which evaluate physical (MSIS-29_phys_; 20 items) and psychological (MSIS-29_psych_; 9 items) impairments. The Twelve-Item MS Walking scale (MSWS-12) was used to evaluate patient-reported walking disability. The SF-36 scale assessed eight different patient domains: physical functioning, role physical, bodily pain, general health, vitality, social functioning, role emotional, mental health, and two global scores (physical and mental). Higher scores on EDSS, MSIS-29, and MSWS-12 indicated greater patient disability.

The total scores from both the MSIS-29 and MSWS-12 were transformed into a score from 0 to 100 for further evaluation.

### 4.3. Statistical Analysis

Descriptive statistics were performed for each group and for the total sample. Means, standard deviations (SD), and 95% confidence intervals (CI) were calculated. The means between the groups and means for the total sample at baseline and at six-week follow-up were compared with a t-test, corrected for multiple comparisons. *p* values of ≤0.05 were considered to be significant.

The Pearson’s correlation coefficient was calculated between bodily pain and age, duration of MS, EDSS, MSIS-29 _phys_, MSIS-29_psych_, MSWS-12, and the SF-36 categories (emotional role functioning, physical role functioning, vitality, general health perceptions, social role functioning, physical functioning, and mental health) at relapse onset and six weeks later.

## 5. Conclusions

Pain was common in our cohort of MS patients; it worsened following relapse and was still significant six weeks after treatment with corticosteroids, where it was albeit less severe than at relapse onset. Patients with a greater number of prior relapses and greater disability experienced greater pain at relapse onset. Pain was associated with a poorer physical and psychological well-being and quality of life.

Our data indicate a bi-directional relationship between relapse-related pain and physical and psychological well-being, a finding that argues for an interdisciplinary approach to treating MS patients during a relapse.

## Figures and Tables

**Table 1:** Patient demographics at relapse and at six-week follow-up.

	With Pain		No Pain		Total	
At relapse onset ^a^						
N	86 (74%)		31 (26%)		117 (100%)	
Female	71 (18%)		21 (61%)		92 (79%)	
	No./Mean (SD)	CI	No./Mean (SD)	CI	Mean (SD)	CI
Age	37.8 (7.8)	36.1–39.4	39.9 (11.2)	35.9–44.0	38.3 (8.8)	36.7–39.9
Number of relapses in last 2 years	2.3 (1.4)	2.0–2.6	1.3 (1.0)	0.9–1.7	2.0 (1.4)	1.8–2.3
Years since diagnosis	6.7 (6.2)	5.4–8.1	8.2 (8.0)	5.2–11.2	7.1 (6.7)	5.9–8.4
EDSS	5.0 (1.3)	4.7–5.3	4.7 (1.5)	4.1–5.2	4.9 (1.4) ***	4.6–5.1
MSIS-29_PHY_	67.2 (20.0) ***	62.9–71.5	41.0 (25.8)	31.6–50.5	60.3 (24.5) ***	55.8–64.7
MSIS-29_PSY_	61.7 (22.8) ***	56.8–66.6	36.5 (22.6)	27.9–45.1	55.3 (25.2) ***	50.7–60.0
MSWS-12	74.6 (23.9)	69.4–79.7	49.7 (32.1)	37.7–61.7	68.1 (28.3) ***	62.9–73.3
SF-36 categories						
Emotional role functioning	37.2 (43.8)	27.8–46.6	53.8 (46.9)	36.6–71.0	41.6 (45.0)	33.3–49.8
Physical role functioning	8.1 (22.2) ***	3.4–12.9	29.0 (37.7)	15.2–42.9	13.7 (28.5) **	8.5–18.9
Bodily pain	37.6 (21.6) ***	32.9–42.2	95.9 (7.1)	93.3–98.5	53.0 (32.0) **	47.2–58.9
Vitality	27.1 (18.6) ***	23.1–31.0	43.2 (25.5)	33.9–52.6	31.3 (21.8) **	27.4–35.3
General health perceptions	41.7 (21.5) ***	37.0–46.4	61.1 (23.5)	52.5–69.7	46.9 (23.6)	42.6–51.3
Social role functioning	37.4 (26.1) ***	31.8–43.0	56.9 (27.2)	46.9–66.8	42.5 (27.7) ***	37.5–47.6
Physical functioning	32.9 (25.2)	27.5–38.3	57.7 (33.8)	45.3–70.1	39.5 (29.7) *	34.0–44.9
Mental health	53.3 (21.6) ***	48.6–57.9	68.0 (19.7)	60.8–75.2	57.2 (22.1) **	53.1–61.2
Six weeks after relapse onset ^b^						
N	73 (65%)		39 (35%)		112 (100%)	
Female	59 (53%)		28 (25%)		87 (78%)	
Age	38.7 (8.7)	36.6–40.7	38.8 (8.9)	35.9–41.6	38.3 (8.8)	36.7–39.9
Number of relapses in last 2 years	2.0 (1.5)	1.7–2.4	1.9 (1.3)	1.5–2.3	2.0 (1.4)	1.8–2.3
Years since diagnosis	6.7 (5.9)	5.3–8.1	7.9 (8.1)	5.2–10.6	7.1 (6.7)	5.9–8.4
EDSS	3.9 (1.7)	3.5–4.3	3.8 (2.1)	3.1–4.5	3.9 (1.8)	3.5–4.2
MSIS-29_PHY_	45.1 (23.0) ***	39.6–50.7	26.7 (25.3)	18.5–34.9	38.4 (25.4)	33.5–43.3
MSIS-29_PSY_	42.7 (26.0) ***	36.4–49.0	26.3 (22.8)	18.9–33.7	36.7 (26.1)	31.7–41.7
MSWS-12	53.9 (26.8) ***	47.6–60.1	35.1 (32.5)	24.5–45.6	47.3 (30.2)	41.7–53.0
SF-36 categories						
Emotional role functioning	40.0 (42.9) ***	29.9–50.0	70.9 (42.7)	57.1–84.8	50.7 (45.2)	42.3–59.2
Physical role functioning	14.9 (26.8) ***	8.6–21.2	48.7 (43.7)	34.6–62.9	26.8 (37.2)	19.8–33.8
Bodily pain	46.9 (19.9) ***	42.2–51.5	93.7 (8.0)	91.1–96.4	63.2 (28.0)	58.0–68.4
Vitality	34.5 (19.5) ***	29.9–39.0	49.6 (24.1)	41.7–57.4	39.8 (22.3)	35.6–44.0
General health perceptions	46.2 (23.7) **	40.6–51.9	57.7 (22.9)	50.3–65.2	50.3 (24.0)	45.8–54.9
Social role functioning	47.6 (27.8) ***	41.1–54.1	74.7 (26.0)	66.2–83.1	57.0 (30.0)	51.4–62.6
Physical functioning	41.2 (22.5) ***	35.9–46.5	60.0 (33.8)	49.0–71.0	47.8 (28.3)	42.5–53.1
Mental health	59.5 (20.2) ***	54.7–64.3	74.0 (21.8)	66.9–81.0	64.6 (21.8)	60.5–68.8

Abbreviations: No.—number of patients; SD—standard deviation; CI—95% confidence interval; EDSS—Expanded Disability Status Scale; MSIS-29—Multiple Sclerosis Impact Scale transformed score for physical and psychological part; and MSWS-12—Twelve-Item MS Walking scale transformed score. ^a^ Data were missing for number of relapses in the last two years for one patient with bodily pain, one for number of years since diagnosis, two for MSIS-29_PSY_, and one for MSWS-12. Data were missing for five patients without pain for number of years since diagnosis, and two patients for general health perceptions. ^b^ Data were missing for one patient with bodily pain for number of relapses in the last two years, for physical role functioning, and for vitality; for two patients for number of years since diagnosis and mental health; for three for general health perceptions; for five for EDSS, MSIS-29_PHY_, and MSIS-29_PSY_. Data were missing for two patients without pain for years since diagnosis, and EDSS. Significant differences between patients with and without bodily pain or between all patients at the relapse onset and at the 6-week follow up. * *p* < 0.05; ** *p* < 0.01; and *** *p* ≤ 0.001.

**Table 2:** Proportion of patients with pain, and severity of pain, at relapse onset and at six-week follow-up.

Severity of Pain	At Relapse Onset (n = 117)	Six Weeks after Relapse Onset (n = 112) ^a^	Changes
none	24 (20.5%)	25 (21.4%)	0.9%
very mild	14 (12.0%)	24 (20.5%)	8.5%
mild	14 (12.0%)	23 (19.7%)	7.7%
moderate	33 (28.2%)	23 (19.7%)	−8.5%
severe	23 (19.7%)	15 (12.8%)	−6.8%
very severe	9 (7.7%)	2 (1.7%)	−6.0%

^a^ Data were missing for 5 patients at six-week follow-up.

**Table 3:** Correlation of bodily pain with demographic factors and physical and psychological impact of relapse.

	At Relapse Onset ^a^		Six Weeks after Relapse Onset ^b^	
	R	*p*	r	*p*
Age	0.043	0.649	0.051	0.594
Number of relapses in last 2 years	−0.248	0.007	−0.097	0.313
Years since diagnosis	0.047	0.624	0.103	0.281
EDSS	−0.118	0.206	−0.071	0.473
MSIS-29_PHY_	−0.506	<0.001	−0.522	<0.001
MSIS-29_PSY_	−0.472	<0.001	−0.445	<0.001
MSWS-12	−0.417	<0.001	−0.379	<0.001
SF-36 categories				
Emotional role functioning	0.243	0.008	0.339	<0.001
Physical role functioning	0.381	<0.001	0.480	<0.001
Vitality	0.379	<0.001	0.503	<0.001
General health perceptions	0.378	<0.001	0.354	<0.001
Social role functioning	0.385	<0.001	0.536	<0.001
Physical functioning	0.418	<0.001	0.414	<0.001
Mental health	0.325	<0.001	0.400	<0.001

Abbreviations: r—Pearson’s correlation coefficient; EDSS—Expanded Disability Status Scale; MSIS-29—Multiple Sclerosis Impact Scale transformed score for physical and psychological part; and MSWS-12—Twelve-Item MS Walking scale transformed score. ^a^ Data were missing for number of relapses in the last two years for one patient with bodily pain, one for number of years since diagnosis, two for MSIS-29_PSY_, and one for MSWS-12. Data were missing for five patients without pain for number of years since diagnosis, and two patients for general health perceptions. ^b^ Data were missing for one patient with bodily pain for number of relapses in the last two years, for physical role functioning, and for vitality; for two patients for number of years since diagnosis and mental health; for three for general health perceptions; and for five for EDSS, MSIS-29_PHY_, and MSIS-29_PSY_. Data were missing for two patients without pain for years since diagnosis, and EDSS.

## Data Availability

Due to ethical restrictions data are not available.
